# materialmodifier: An R package of photo editing effects for material perception research

**DOI:** 10.3758/s13428-023-02116-2

**Published:** 2023-05-10

**Authors:** Hiroyuki Tsuda, Hideaki Kawabata

**Affiliations:** 1 Faculty of Psychology, Doshisha University, Kyoto, Japan; 2 Department of Psychology, Faculty of Letters, Keio University, Tokyo, Japan

**Keywords:** Material perception, Image processing, R package, Open-source software

## Abstract

In this paper, we introduce an R package that performs automated photo editing effects. Specifically, it is an R implementation of an image-processing algorithm proposed by Boyadzhiev et al. (2015). The software allows the user to manipulate the appearance of objects in photographs, such as emphasizing facial blemishes and wrinkles, smoothing the skin, or enhancing the gloss of fruit. It provides a reproducible method to quantitatively control specific surface properties of objects (e.g., gloss and roughness), which is useful for researchers interested in topics related to material perception, from basic mechanisms of perception to the aesthetic evaluation of faces and objects. We describe the functionality, usage, and algorithm of the method, report on the findings of a behavioral evaluation experiment, and discuss its usefulness and limitations for psychological research. The package can be installed via CRAN, and documentation and source code are available at https://github.com/tsuda16k/materialmodifier.

## Introduction

**Fig. 1:**
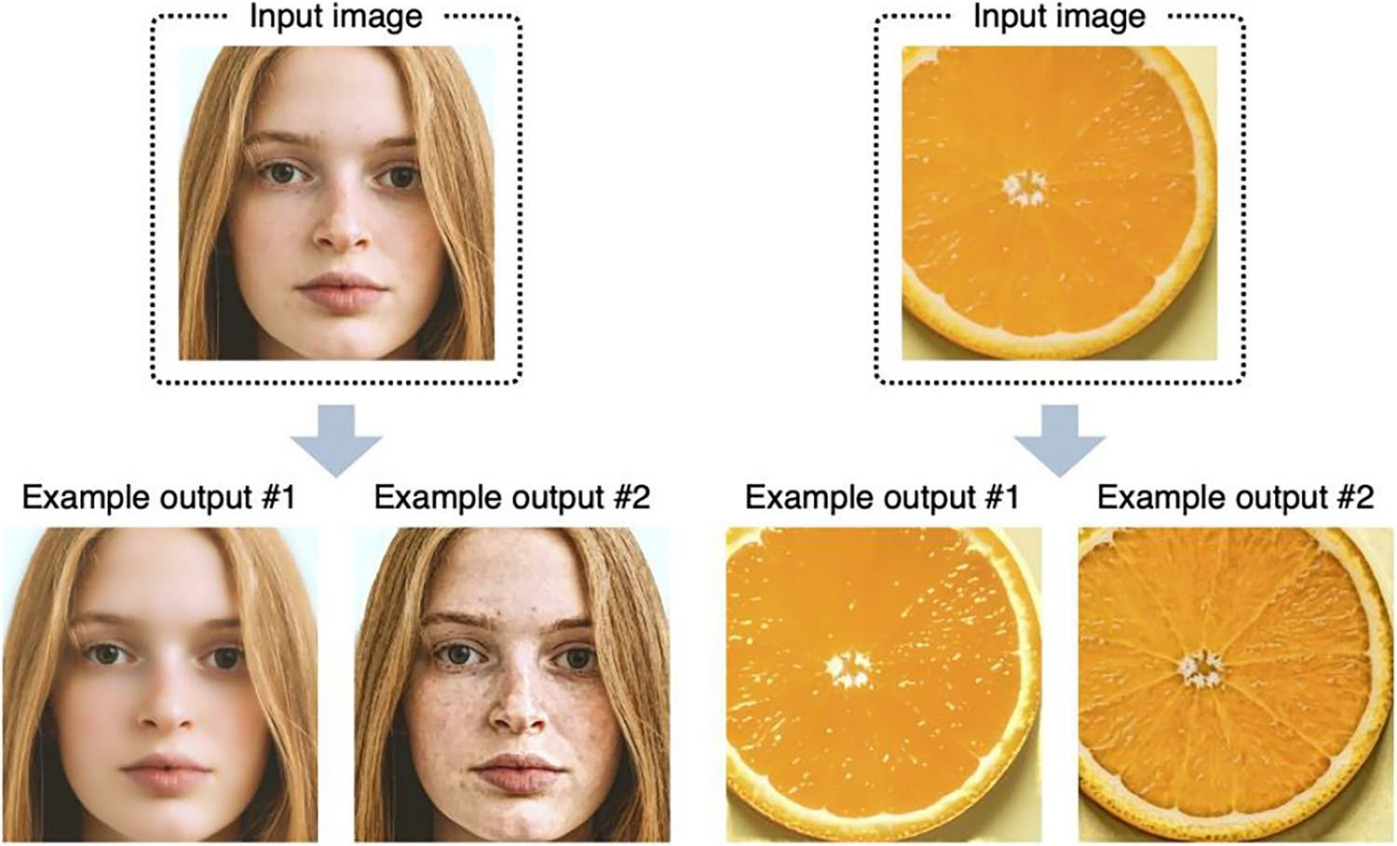
By using the materialmodifier package in R, the user can modify the appearance of objects in photographs. For example, they can make skin smoother or make marks or blemishes more visible; enhance the gloss of food or make it look wilted

Material perception is a rapidly growing research area in vision science today (Fleming, 2017; Komatsu & Goda, 2018; Spence, 2020) and it is relevant to a wide range of human cognition and behaviors (as described below). To study material perception, we need a set of controlled images for stimuli, such as images with high and low roughness. However, unlike basic visual features such as color and lightness, controlling specific material properties of objects in photographs is an intricate endeavor. To alleviate this situation, we created an R package called *materialmodifier* that can be used to modify the surface properties of objects such as gloss and roughness (Fig. 1). This method was proposed by Boyadzhiev et al. (2015), and we implemented it in the R package to make it accessible to psychologists. Before going into the details of the package, we briefly describe recent research trends in material perception to provide some background on our contribution.

People easily perceive and recognize materials in their daily lives and can identify categories of materials quickly and reliably (Fleming et al., 2013; Sharan et al., 2014). People can also distinguish subtle differences in certain material properties, such as the degree of surface roughness and gloss (Fleming, 2017). This visual ability is important for diagnosing the freshness of food or the health of a person based on the condition of their skin. Despite the subjective ease of material perception, achieving stability therein is a computationally challenging problem because retinal input for objects of the same material can vary greatly depending on illumination and the surface shape of the object (Anderson, 2020; Chadwick & Kentridge, 2015; Fleming, 2014). Recent theories suggest that the brain achieves material perception not through inverse-optics computation but through statistical inference based on internal image models (Fleming, 2014; Fleming & Storrs, 2019). From this perspective, systematic manipulation of image features and examining their effects on perception is an effective approach to understanding the mechanisms of material perception (Nishida, 2019).

Material perception is interesting because of its relevance to a wide range of human cognition and behaviors. For instance, material perception has been related to the perception of the freshness of foods (Arce-Lopera et al., 2013; Péneau et al., 2007), judgments of facial impressions from skin conditions (Fink et al., 2006; Fink & Matts, 2008; Jaeger et al., 2018; Nkengne et al., 2008; Stephen et al., 2009), action planning for touching objects and walking on slippery floors (Adams et al., 2016; Joh et al., 2006; Lesch et al., 2008), pathogen detection (Iwasa et al., 2020), product packaging design (Di Cicco et al., 2021), and aesthetic appreciation of textures (Stephens & Hoffman, 2016), paintings (Di Cicco et al., 2020), and sculptures (Schmidt, 2019). Furthermore, studies have explored how material perception contributes to other cognitive domains, such as memory (Tagai et al., 2016; Tsuda et al., 2020) and multisensory perception (Fujisaki, 2020; Spence, 2020).

Despite its wide importance in cognition and behavior, studies on material perception are relatively limited in size and scope. One of the reasons for this may be the difficulty of creating a set of controlled stimuli that differ in certain material properties. There are image databases of materials and textures that are useful for psychological research (Lagunas et al., 2019; Sawayama et al., 2019; Serrano et al., 2021; Sharan et al., 2014; van Zuijlen et al., 2021). However, we often need a new image set tailored for specific research purposes. In such cases, images are created either by manual photo editing, using software such as Photoshop and GIMP (Fink & Matts, 2008; Jaeger et al., 2018), taking photographs of objects (Motoyoshi et al., 2007), or using computer graphics rendering (Fleming et al., 2003). In any of these circumstances, a decent amount of time, effort, or technical expertise is required. Moreover, the manual editing of photographs suffers from the low reproducibility of the image production process.

In this study, we implemented the image processing algorithm proposed by Boyadzhiev et al. (2015) as an R package. It is one of the image-based material editing methods: a heuristic method that manipulates image features that are associated with human material perception. Specifically, it decomposes an image into spatial-frequency subbands (i.e., images representing specific spatial frequency information of the input image) and changes the input image’s appearance by manipulating (boosting/reducing) the energy of specific subbands therein (details are given in the Algorithm section). Although the algorithm for this method is simple and heuristic, it is effective and compelling for the following reasons. The human early visual system represents visual information in spatial frequency and orientation selective channels (Blakemore & Campbell, 1969), and a computational model of early vision based on spatial frequency decomposition explains human contrast detection/discrimination well (Schütt & Wichmann, 2017). Spatial frequency subband statistics are associated with the perception of the material properties of objects, e.g., gloss (Dror et al., 2004; Kiyokawa et al., 2021; Motoyoshi & Matoba, 2012). Manipulating the energy of specific spatial frequency subbands and their correlations can effectively modify the perceptual attributes of textures (Giesel & Zaidi, 2013; Portilla & Simoncelli, 2000). Therefore, image-editing methods based on the manipulation of an image’s spatial frequency characteristics can be thought of as effectively exploiting the mechanism by which the human visual system encodes information about the external world. In this regard, Boyadzhiev et al.’s (2015) method is interesting not only as an image-editing tool, but also as a model of vision.

In the following sections, we describe the functionality and usage of the package, illustrate the behavior of the algorithm with a number of image examples, and explain how the algorithm works. We also report on an experiment that examined how face and food images edited using this method affect viewers’ perception of the material properties of said faces and food, and discuss its usefulness and limitations in psychological research.

## Functionality and usage of the package

This section describes the features of the package and how to use them. Note that detailed instructions and practical tips for using the package, as well as the source code, are provided on our GitHub page (https://github.com/tsuda16k/materialmodifier). The basic procedure to use this package is as follows.

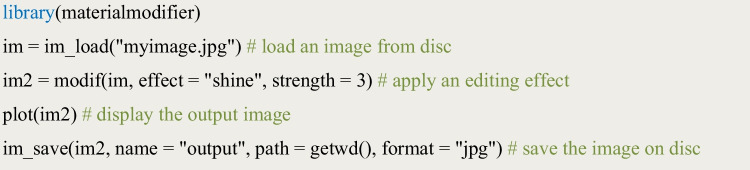



You can load an image from the disk with the im_load() function, and apply a material editing effect with the modif() function. The *effect* argument of the modif function specifies the type of material editing effect applied (explained below), and the *strength* argument determines the strength of the effect. The plot() function can be used to display an image. To save an image on disk, use the im_save() function, specifying the name of the output image file and the path where the image will be saved. You can load/save images in jpg/png/bmp format.

**Fig. 2:**
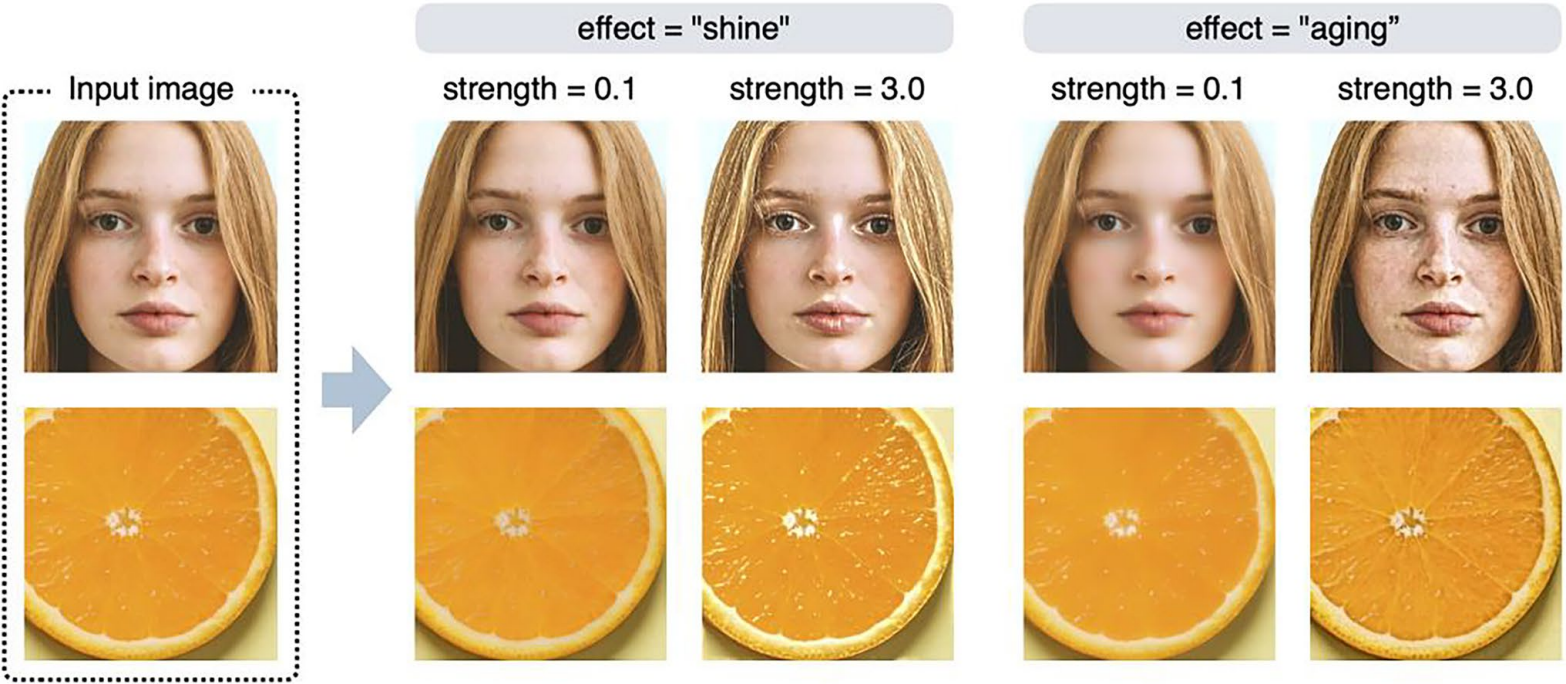
Example outputs of the shine and aging effects. The strength parameter controls the strength of the editing effect

Figure 2 shows example outputs of the *shine* and *aging* effects. The shine effect manipulates very bright elements in the high spatial frequency bands in the input image (e.g., highlights and gloss), and the aging effect manipulates local dark elements (e.g., stains and blemishes). The higher the value of the strength parameter, the stronger the editing effect (the features are emphasized). If the value of the strength parameter is less than 1, the opposite effect will occur, e.g., the gloss will be weakened or blemishes will be reduced.

**Fig. 3:**
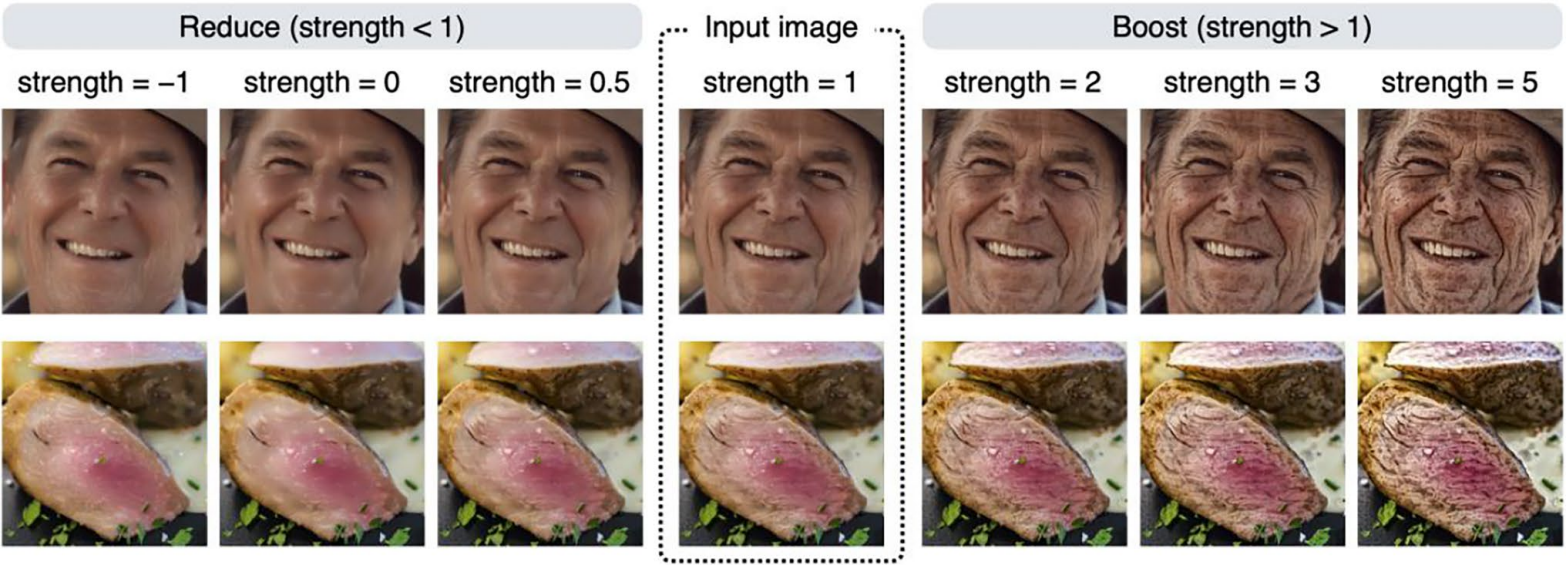
The effect of the strength parameter is examined using the aging effect. If the value of the strength parameter is greater than 1, a boosting effect occurs; if it is less than 1, a reducing effect occurs

Using the aging effect as an example, the effect of the strength parameter is examined in more detail in Fig. 3. If the value of the strength parameter is greater than 1, a boosting effect that increases the stains/blemishes occurs; if it is less than 1, a reducing effect that decreases the stains/blemishes occurs. To achieve a boosting effect, a strength value of 1.5 to 4 usually yields reasonable results. The strength parameter can be a negative value, but in most cases, setting a negative value will produce unrealistic results (e.g., contrast reversal; see also Fig. 5). Note that if the strength parameter is 1, no effect occurs, and the input image is returned unchanged. This is because this parameter is a multiplication factor for the image feature being manipulated (a detailed description of this parameter is given in the Algorithm section).

You can also apply multiple editing effects simultaneously. For example, you can simultaneously apply the shine and the aging effects as follows.

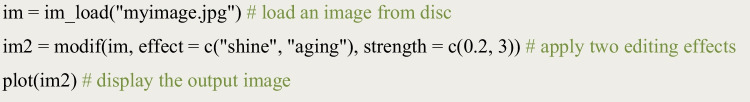



This command simultaneously applies a shine effect of strength = 0.2 and an aging effect of strength = 3, resulting in a less shiny and more blemished image. This procedure is the same as the one used to create output example #2 in Fig. 1. Although you can obtain almost the same result (but not identical, because the first process changes the input image for the second process) by applying each effect in turn (e.g., applying an aging effect to the output of a shine effect), we recommend doing them in a single line, as in the example above, because it saves time needed for image processing. The order of effect names specified in the effect argument does not affect the result; effect = c("shine", "aging") and effect = c("aging", "shine") produce identical results.

**Fig. 4:**
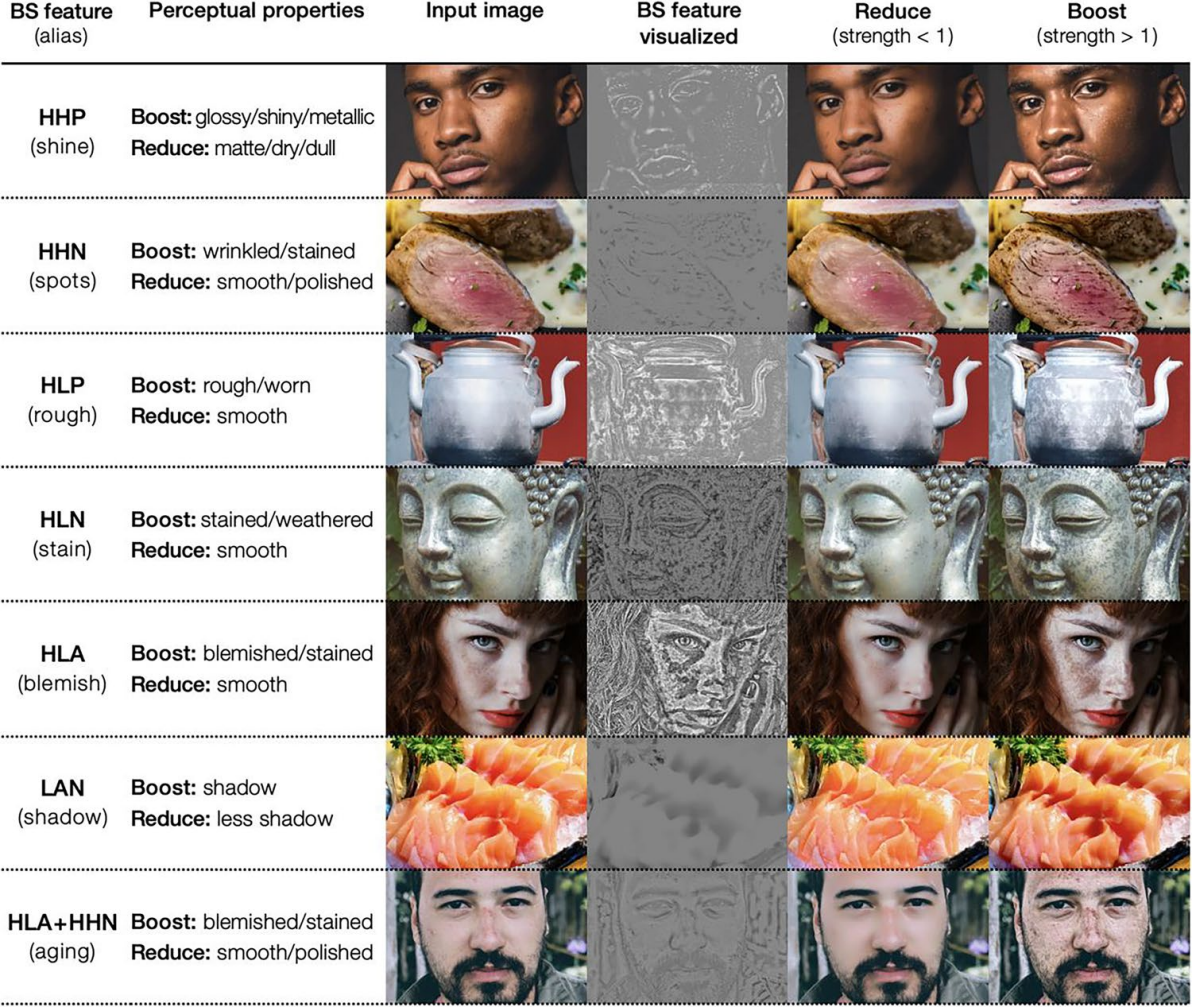
Visual summary of image editing effects. By specifying the name of an effect (or BS feature), the algorithm detects that feature in the input image and modifies the appearance of the input image by reducing or boosting the feature. Note that the aging effect controls both HLA and HHN features. See the main text for the definition of BS features

This package has several other effects in addition to the shine and aging effects. The available effects are shine, spots, rough, stain, blemish, shadow, and aging. A visual summary of these effects is shown in Fig. 4. The first column of the figure shows the name of each effect, and the second column shows the perceptual features controlled by that effect. The third and subsequent columns show the input and output images.

Figure 4 contains a column labeled “BS (band-sifting) feature”; this is an important term related to the image processing algorithm (briefly, an image component to be manipulated, extracted from the input image based on a certain criterion). The algorithm achieves image editing effects by decreasing or increasing the weights of the BS features in the input image. The effect names, such as shine and spots, are aliases for these BS features. The input to the effect argument of the modif function can also be the BS feature names.

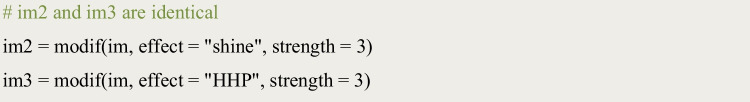



Since it is easier to know what kind of editing effect will be achieved if there is an alias, our implementation allows the user to specify image editing by alias as well as by BS feature name.

**Fig. 5:**
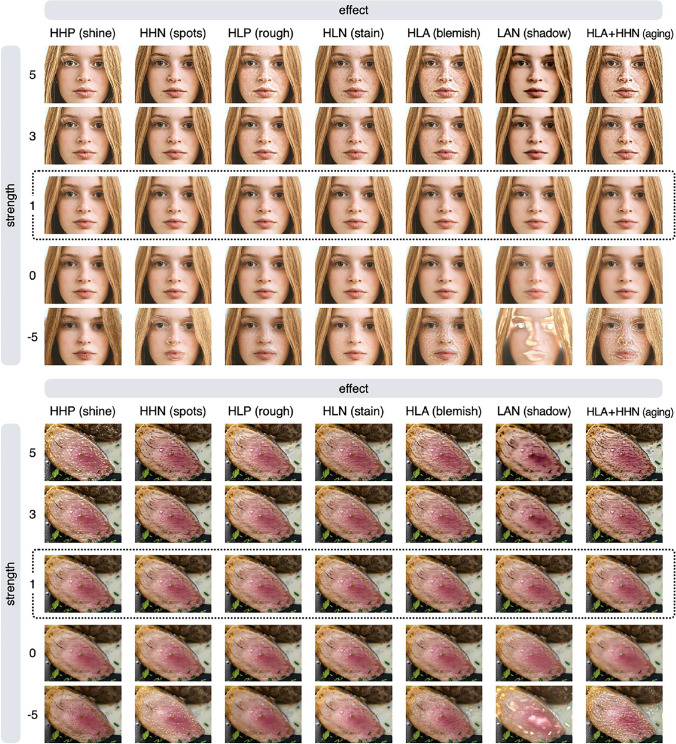
Summary of image processing results with different strength values for each effect. The image inside the dotted line is the input image. Each image in the row with a strength value of −5 has an unnatural appearance (contrast reversal) and is not suitable for use as a stimulus, but it is useful to visualize what feature in the input image is manipulated by each effect

To understand the nature of each image editing effect better, it is helpful to compare the results of all editing effects on a single image. Figure 5 summarizes the results of the editing effects on face and food images (note that the image in the row with a strength value of 1 is the input image). By comparing the images in the rows with large values of the strength parameter, it is easier to see the characteristics of each effect.

Setting a negative value for the strength parameter often results in an unnatural image (see images in the bottom rows of Fig. 5), but using a large negative value for the strength parameter makes it easier to compare which areas are affected by each effect. For example, the rough effect and the blemish effect produce similar results, but if you compare the images in the row with a strength value of −5, you can clearly see that they are not identical. Technically, the blemish effect is equivalent to giving both the rough and stain effects at the same time. To acquire a more formal understanding of these properties, we need to know more about the specifics of how image processing algorithms work.

**Fig. 6:**
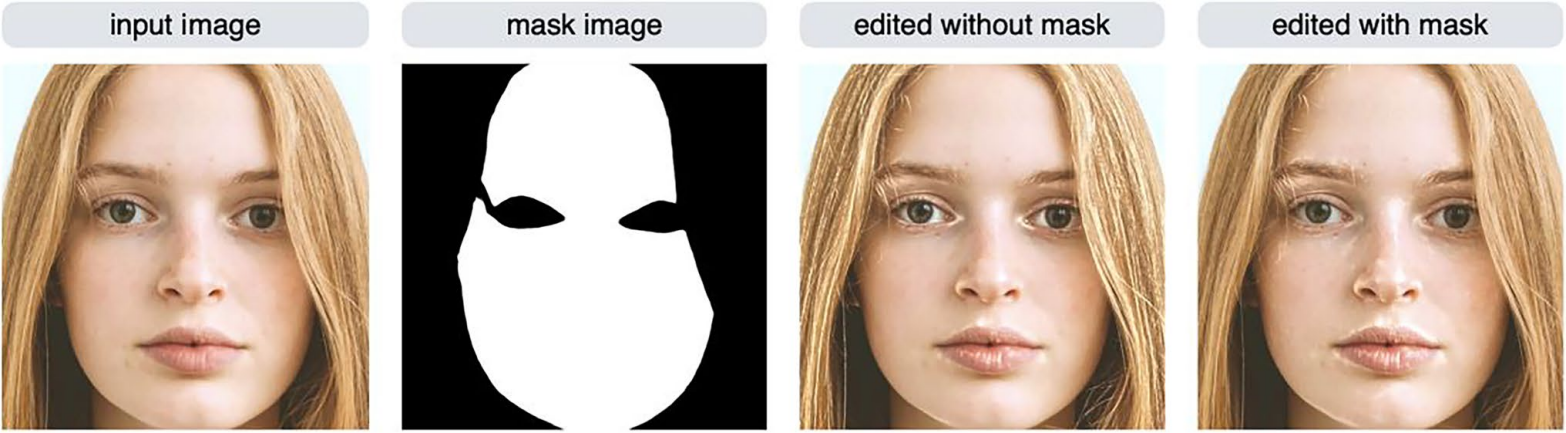
Editing only specific areas in an image using a mask image. The results of the HHP (shine) effect (strength = 3) with and without using a mask image are shown

By default, the modif function targets the entire image for editing. However, in some situations, you may want to edit only certain objects or areas of the image (for example, you may want to edit only the skin area of a portrait). By using a mask image, you limit editing to certain areas within an image. To use this feature, you need to prepare a mask image of the same size as the input image you wish to edit. The mask image contains the area to be edited—white in color—and the rest of the image—which is black. The mask image does not have to be a binary image; gray can be present (the intensity of the gray will be used to alpha blend the input image with the edited image). For example, the mask image representing the skin region of a face image is shown in Fig. 6.

The image edited without using a mask image has an increased gloss not only on the skin, but also on the hair and eyes. On the other hand, the image edited using a mask image has increased gloss only in the skin area. To use the masking feature, a mask image in the mask argument of the modif function must be specified.

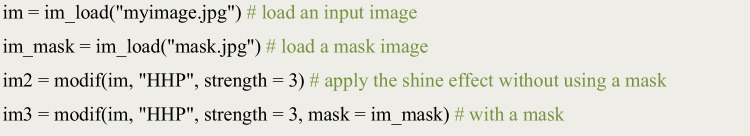



## Algorithm

In this section, we will describe the image processing algorithm in detail. First, there are two points to note. First, the user does not necessarily need to understand the details of the algorithms to use this package. In fact, as we have seen, it is possible to perform image processing by simply specifying the name of the effect and the strength parameter. However, by reading this section, users will have a better understanding of the behavior of this package and will be able to use it in an advanced way. Secondly, this paper will explain the algorithm at a conceptual level, which would be appropriate for the average psychologist. The technical and mathematical aspects are explained in the original paper (Boyadzhiev et al., 2015).

**Fig. 7:**
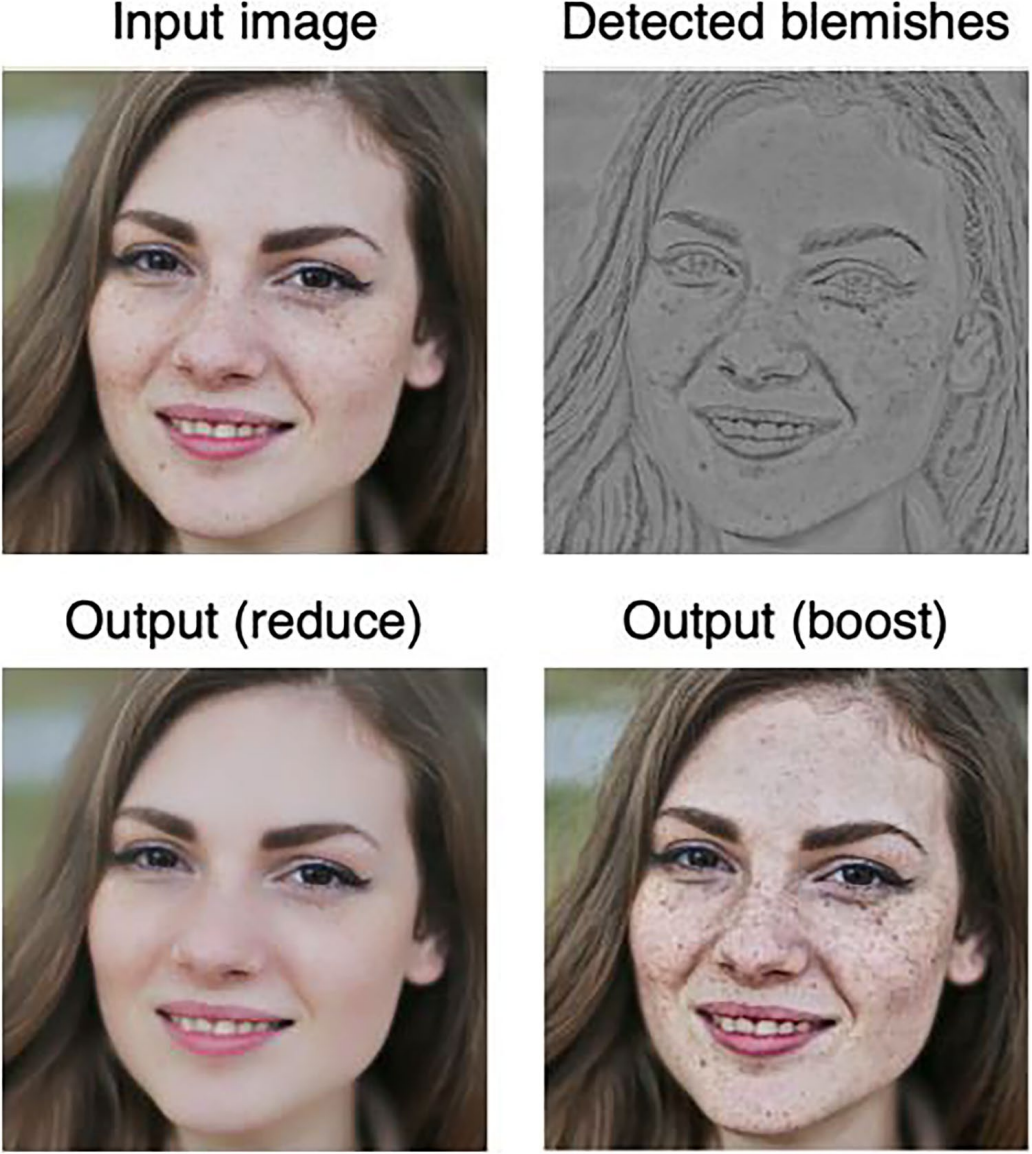
The central idea of the material editing algorithm. Pixels corresponding to image features that are to be manipulated (e.g., blemishes; top right) are extracted from the lightness channel of the input image based on specific criteria. By decreasing or increasing the lightness value of those pixels, the appearance of the object surface in the input image is controlled (bottom left and bottom right)

Boyadzhiev et al. (2015) proposed an image-based material editing method called “band-sifting decomposition.” It extracts and controls a variety of perceptual properties of images such as gloss, roughness, and blemishes based on a combination of image processing procedures. How this algorithm modifies the surface appearance of an input image is shown in Fig. 7, using the control of blemishes as an example. Pixels corresponding to image features that are to be manipulated (e.g., blemishes) are extracted from the lightness channel of the input image based on specific criteria. By decreasing or increasing the lightness value of the pixels in the feature image (Fig. 7, top right), the appearance of the object surface in the input image is controlled.

**Fig. 8:**
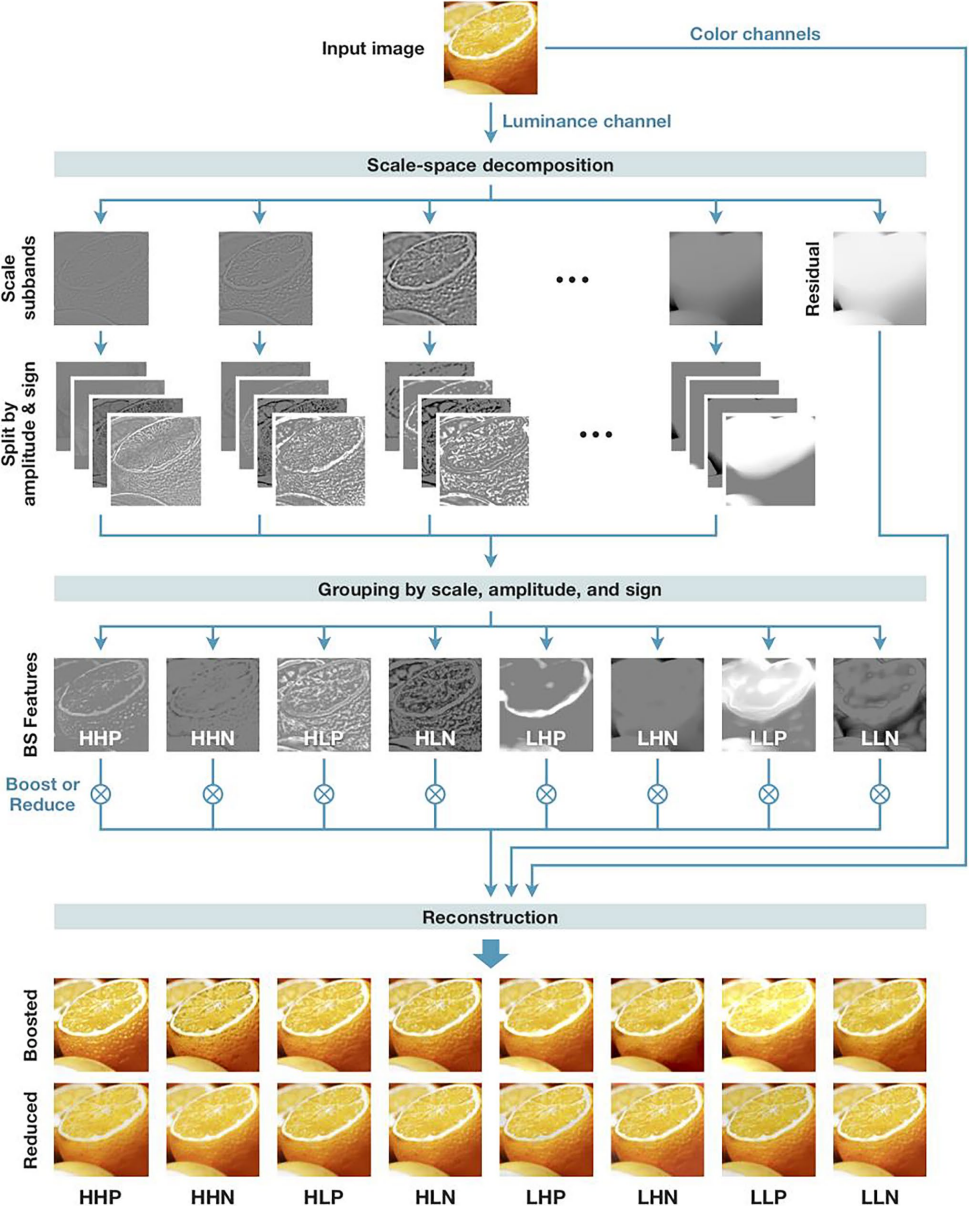
The image processing flow for material editing. The L channel of an input image is decomposed into multiple component images based on three criteria: scale (spatial frequency), amplitude (low or high contrast), and sign (sign of pixel value: positive or negative). The component images are assigned to eight groups based on scale, amplitude, and sign. The images are then combined (added together) within each group, resulting in eight images that we call BS (band-sift) features. These images represent different aspects of the perceptual quality of the input image, which can be used to control the appearance of objects. See the main text for a definition of each abbreviation (e.g., HHP)

The overall flow of image processing is summarized in Fig. 8. The input image is first converted to the CIELAB color space. We only process the lightness channel (L channel) and keep the color channels intact. The L channel is log-transformed and then decomposed into “scale subbands”; each subband image represents the lightness information at a given scale (or spatial frequency). As in Boyadzhiev et al. (2015), we employed the “guided filter” (He et al., 2010) to perform scale decomposition. This procedure is a type of band-pass filtering, which decomposes the image based on spatial frequency. This decomposition differentiates between small-scale elements, such as blobs and wrinkles, and large-scale gradients, such as shading and shadows. The number of subband images is determined by the resolution of the input image: if the shorter side of the input image has N pixels, then log_2_N − 1 subband images are produced (e.g., if N = 512, then 8 subband images are produced). The decomposition also produces a low-frequency residual.

Each subband image is further decomposed into four images based on the amplitude and sign of the pixels in that subband, and this process is the core idea of the algorithm. For amplitude, the standard deviation (1 SD) of pixel values in each subband is used as a threshold between high and low amplitude pixels to separate low- and high-contrast regions of the subband. Each (low/high) amplitude image is then separated by the sign of pixel values, positive or negative; all the negative-value pixels of an amplitude image are set to zero to produce a positive image, and all the positive-value pixels of an amplitude image are set to zero to produce a negative image. Thus, each scale subband is decomposed into four images (high/low amplitude × positive/negative sign; the images of the row labeled “split by amplitude & sign” in Fig. 8).

Next, all the component images (8 scale subbands × 2 amplitudes × 2 signs = 32 images in total, in this example) are grouped by the combination of scale (high or low), amplitude (high or low), and sign (positive or negative). Note that the scale subbands are classified as either high or low frequency (two categories, instead of eight in the original decomposition). If we have N scale subbands, then the first floor (N/2) images are categorized as high frequency and the remaining images as low frequency. This grouping assigns the component images into eight (and always eight, regardless of the resolution of the input image) groups. The images in each group are relatively similar to each other (because they have similar spatial frequencies and belong to the same amplitude and sign group). Finally, the images (pixel values) in each group are added together, resulting in eight images that we call BS features (the images of the row labeled “BS features” in Fig. 8). As in Boyadzhiev et al. (2015), we call each BS feature by the acronym of its grouping criterion. For example, HHP represents the grouping criterion (and the resultant image or BS feature) of the *H*igh spatial frequency, *H*igh amplitude, and *P*ositive sign.

The BS features represent distinct information associated with the perceived material properties of objects in the input image. For example, HHP represents bright (because of their high amplitude and positive sign) and small (because of their high spatial frequency) spots, typically found on wet and glossy surfaces, whereas the HHN (high frequency; high amplitude; negative sign) feature represents small dark blobs that are typical of wrinkles and blemishes in the skin. To amplify gloss, for example, we will boost the HHP feature (i.e., all the pixel values of the HHP image are multiplied by a coefficient greater than 1). To reduce wrinkles, we will reduce the HHN feature (i.e., multiply the HHN image by a coefficient less than 1). Subsequently, all the BS features and the residual image are added together and inverse-log transformed to reconstruct the L channel. The L channel is then combined with color channels and converted back to the standard RGB (red, green, blue) color space to produce the final output (Fig. 8, images in the bottom two rows).

Depending on which BS feature is being manipulated (boosted/reduced), we obtain different material editing effects. The bottom rows of Fig. 8 show the effect of boosting or reducing each BS feature. For example, for the column labeled HHP, the upper image is the result of boosting the HHP feature, while the lower image is the result of reducing the HHP feature. Manipulating BS features with a positive sign (e.g., HH*P* and LL*P*) adjusts the bright areas in the input image, resulting in the editing of features such as glossiness, while manipulating BS features with a negative sign (e.g., HH*N* and HL*N*) adjusts the dark areas in the input image, resulting in the editing of features such as blemishes. Note that not all BS features produce perceptually meaningful changes in the input image. Based on Boyadzhiev et al. (2015) and our observations, we have given aliases (alternative names) to some of the BS features that can be used most effectively for perceptual editing effects. For example, HHP is called the “shine” feature because it can be used to manipulate gloss, and HHN is called the “spots” feature because it can be used to manipulate small stains and wrinkles (see Fig. 4 for the list of aliases).

## Advanced usage of the package

This section describes how to use the package, based on an understanding of how the algorithm works. By using the modif2() function, image manipulation can be performed by specifying a BS feature in detail. There are two ways to do this: using acronyms, or specifying scale, amplitude, and sign separately.

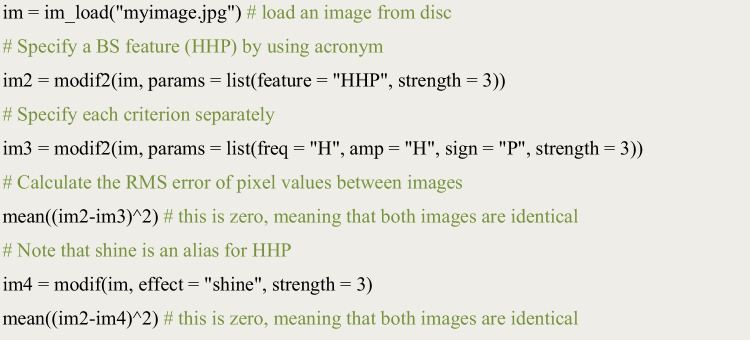



A list of parameters, which specifies a BS feature to be manipulated, is given to the *params* argument. A BS feature can be specified by the *feature* parameter using acronyms. A strength value must also be specified in the list. Instead of using acronyms, each criterion can be specified individually. The *freq* parameter specifies the spatial frequency (“H” for high spatial frequency, “L” for low spatial frequency, or “A” for all frequencies). The *amp* parameter specifies the amplitude (“H” for high amplitude, “L” for low amplitude, or “A” for all (both) amplitudes). The *sign* parameter specifies the sign (“P” for positive sign, “L” for negative sign, or “A” for both signs).

The advantage of specifying the features we want to manipulate using individual criteria is that we have more freedom to specify the scale. As mentioned in the algorithm section, the number of scale-subband images that the algorithm creates is determined by the resolution of the input image. The number of scale subband images for an image can be known using the modif_dim() function. In addition to the number of subband images, this function also outputs the indices of high- and low-scale images. An example is shown below, where the input image is 500 × 500 px in size.

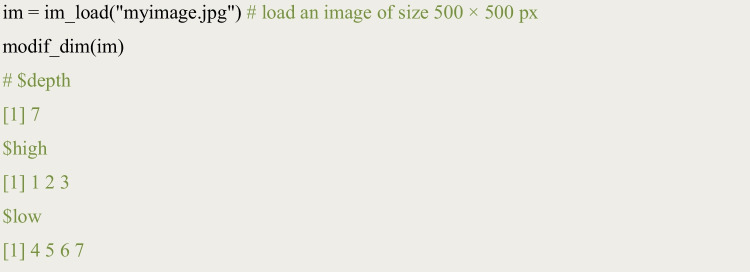



The output of the modif_dim() function shows that the number of subband images to be created from this image is 7; the indices for higher-scale (spatial frequency) images are 1, 2, and 3, and the indices for lower-scale images are 4, 5, 6, and 7. This shows that, in the case of this input image, freq = “H” is equivalent to setting freq = 1:3, and freq = “L” is equivalent to setting freq = 4:7. Therefore, each pair of commands below will produce the same output (but note that which freq corresponds to H/L depends on the resolution of the input image).

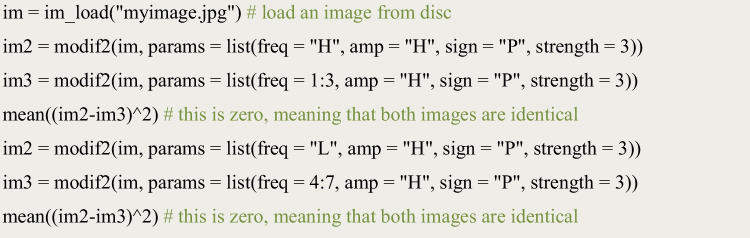



**Fig. 9:**
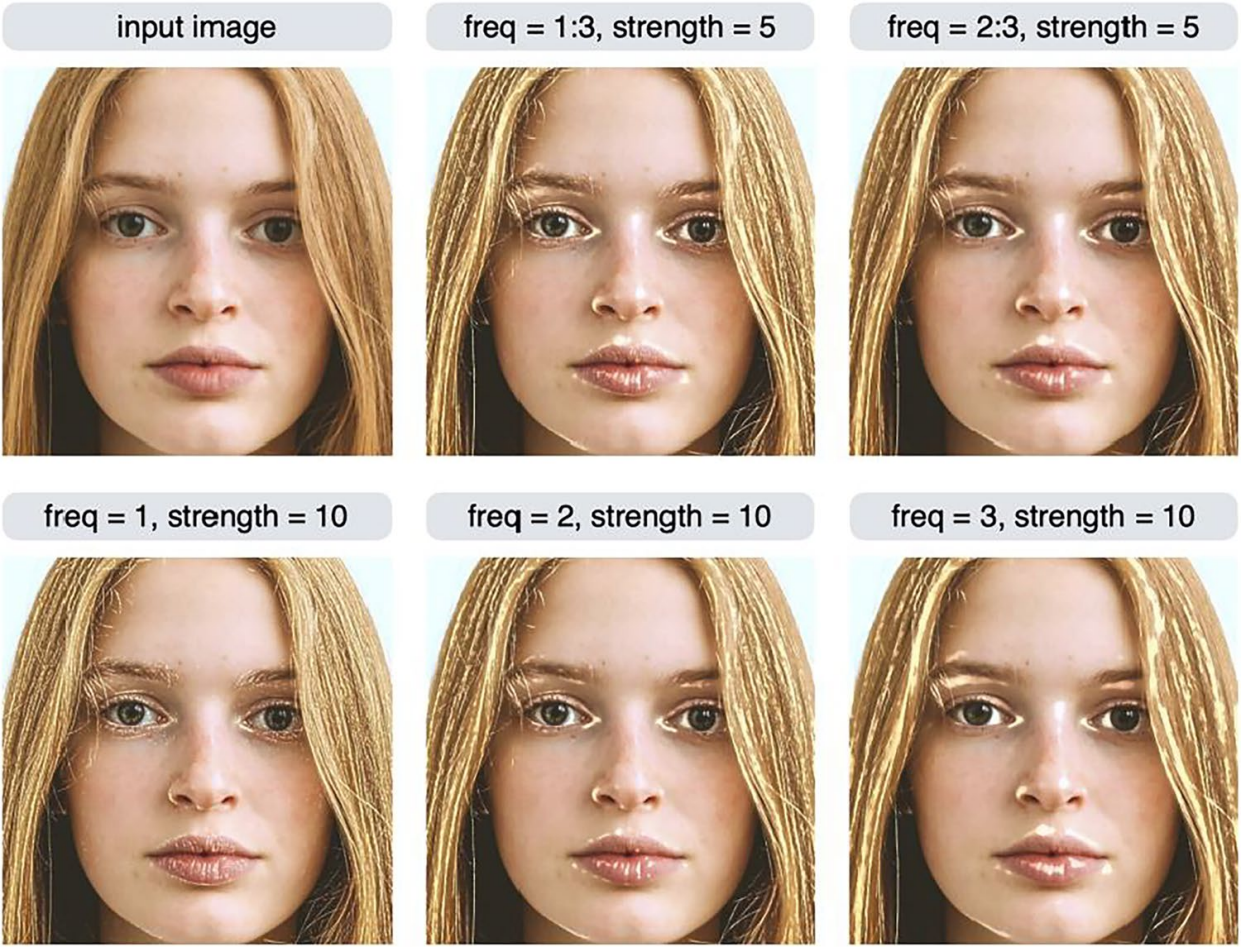
Variants of the HHP (or shine) effect. The modif2 function can be used to specify only one scale subband to be controlled (bottom row), or multiple scale subbands (top middle, top right). The argument of the function was params = list(freq = x, amp = "H", sign = "P", strength = y). The x and y values are shown in the label of each image

What is the advantage of being able to specify the scale to be manipulated in detail? We may want to manipulate only a specific scale, rather than as a high- or low-scale group. To understand this motivation, the result of manipulating individual scales is shown in the bottom row of Fig. 9. When freq = 1, regions such as eyebrows, which are not considered to be skin gloss, are controlled. Image features with the highest spatial frequency (i.e., freq = 1) often reflect the high spatial frequency noise. Therefore, in some cases it is useful to exclude the highest spatial frequency subband when controlling images. The image in the top middle of Fig. 9 has scale = 1, 2, 3, which is identical to specifying freq = "H" (and therefore this command specifies the HHP feature and is equivalent to the shine effect). Some areas of the eyebrows are brighter in this image because the image subband with freq = 1 has been manipulated. On the other hand, the image in the top right of Fig. 9 does not show a manipulation of the freq = 1 region, so there is no change in the brightness of the eyebrows.

**Fig. 10:**
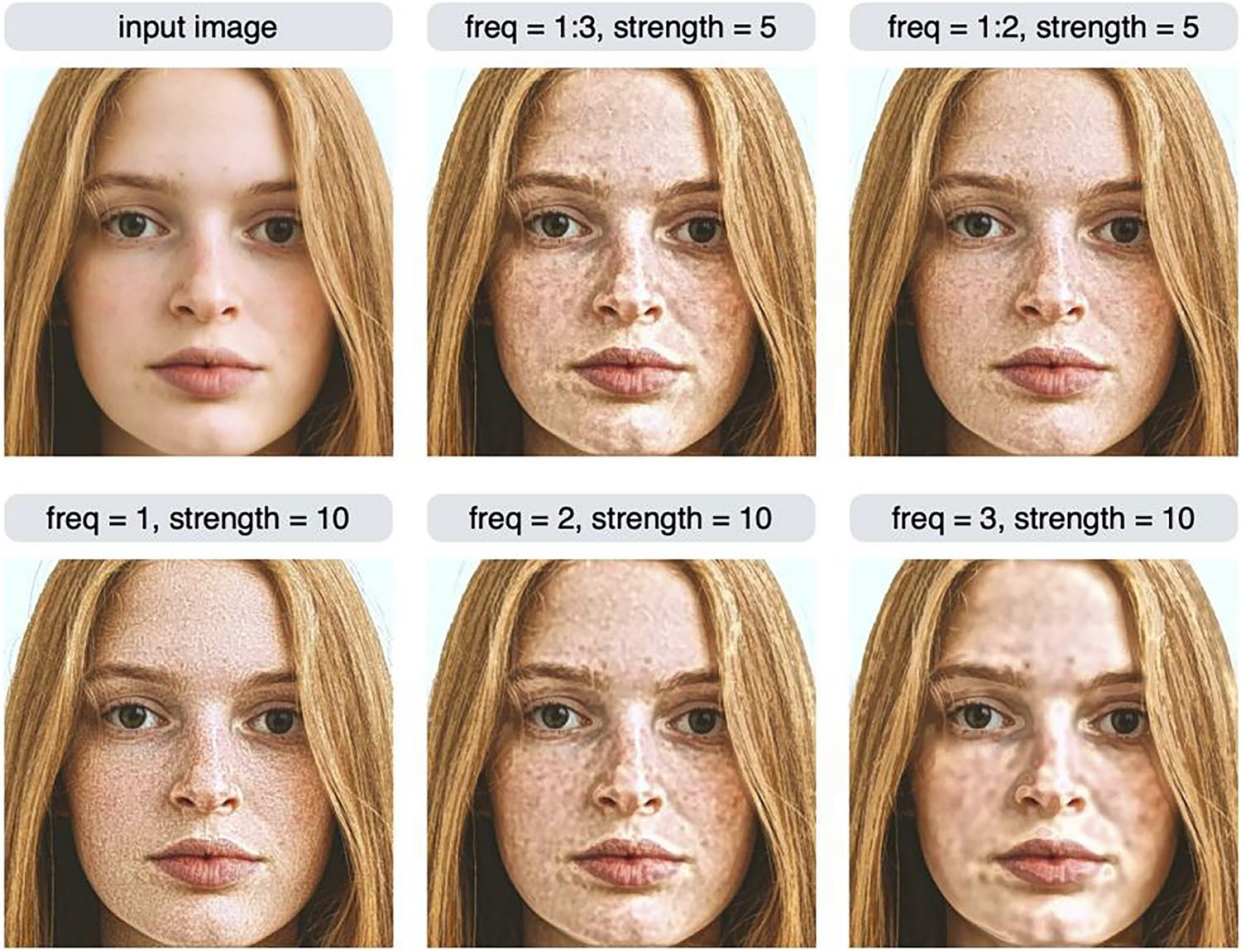
Variants of the HLA (or blemish) effect. The argument of the modif2 function was params = list(freq = x, amp = "L", sign = "A", strength = y). The x and y values are shown in the label of each image. Excluding the third scale resulted in a somewhat different output than the normal stain effect (top right)

Another example of the effect of selecting a particular scale is shown in Fig. 10. These are variants of the HLA (blemish) effect. Instead of specifying freq = "H" (or, equivalently, freq = 1:3), we can specify freq = 1:2 (excluding the third scale), which gives a somewhat different result (Fig. 10, top right) than the normal blemish effect (Fig. 10, top middle).

Recall that in the case of the modif function, it is possible to apply multiple effects simultaneously. A similar approach can be taken with the modif2 function. The following script shows how to apply the spots/rough effect simultaneously using the modif2 function. A list containing several lists of parameters is given as the params argument to the function.

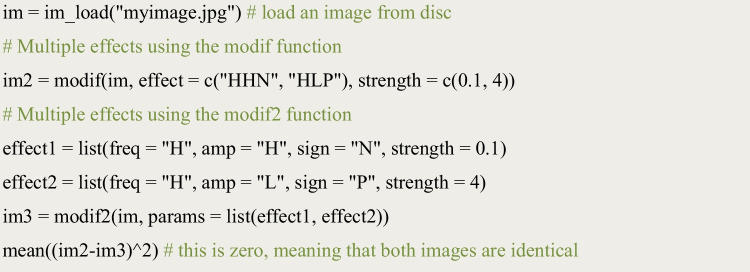



In summary, the modif2 function allows the user to specify the scale subband (i.e., spatial frequency) to be manipulated in more detail than the modif function, and provides greater flexibility in controlling the appearance of images.

## Evaluation experiment

In this section, we report on an online experiment that evaluated how material editing effects can produce perceptual changes in an input image. We created a series of images with different strength parameter values for each editing effect (HHP, HHN, etc.), and conducted an experiment in which we asked participants to rate the perceived material properties of objects, such as gloss and roughness, as well as the naturalness of the images. The material, data, and R scripts for this experiment are available on OSF: https://osf.io/72dqz/.

### Participants

To detect an effect size of Cohen’s d = 0.55 (a difference of 0.5 on a six-point scale; the standard deviation was based on our pilot study) with 90% power (alpha = .05, two-tailed), G*Power (Faul et al., 2007) suggested that we needed 37 participants. In total, 48 participants (female=24, male=24) took part in the experiment. Participants were recruited from psychology classes at Keio University and Doshisha University (M_age_ = 21.1, SD_age_ = 3.8), and they provided informed consent to take part in the study. The study was approved by the Ethics Committees of Keio University (#21026) and Doshisha University (#22070).

### Materials

Two human face images and two food images were used in the experiment. The face images were of an Asian male and a Caucasian female, selected from the Chicago Face Database (Ma et al., 2015; image names: CFD-AM-215-120-N and CFD-WF-003-003-N). The food images were photographs of meat and of a sliced orange; they were selected from public domain licensed images at a stock photos website. Each image was edited with six editing effects (HHP, HHN, HLP, HLN, HLA, and HLA+HHN), and seven strength values (0, 0.25, 0.5, 1, 1.5, 2, and 2.5) were set for face images and 0.25, 0.5, 1, 2, 3, and 4 for the food images. The shadow effect was not included in the experiment because we assumed that shadows are a less important feature in the perception of material attributes tested in the experiment. The reason for varying the range of the strength parameter for the face and food images is that people are more perceptually sensitive to editing manipulations of faces than of non-face objects (Boyadzhiev et al., 2015). For each original image, 42 image variations (6 editing effects × 7 strength values) were created, resulting in 168 stimulus images. Note that images with a strength value of 1 are identical to the original (unedited) image. All images were 500 × 500 px in size and presented at that size on the display.

### Design and procedure

For each stimulus, participants rated six attributes (five material properties and naturalness) on a six-point scale. The material attributes were matte–gloss, dry–wet, opaque–translucent, rough–smooth, and old–young/fresh. These attributes are representative dimensions in skin perception (Otaka et al., 2019) and food perception (Hanada, 2020; Spence et al., 2022). Participants were instructed to rate the naturalness of the images, that is, photorealism. An image was presented in the center of the display and participants responded by pressing a key (there was no time limit on the response). Each image set (42 variations of an original image) and attribute was rated in blocks and in random order. Within each block, images of varying editing effects and strength values were presented in random order. Images with a strength value of 1 were presented twice, while images with other strength values were presented once. The total number of trials was 1152 (4 image contents × 6 rating dimensions × 6 editing effects × (7+1) strength values). Participants completed practice trials prior to the experimental session (a face image and a food image were used in this trial, but not in the experimental session).

### Results

The data of five participants were not recorded (possibly owing to network errors). Thus, 43 participants (20 male and 23 female) were included in the analysis.

**Fig. 11:**
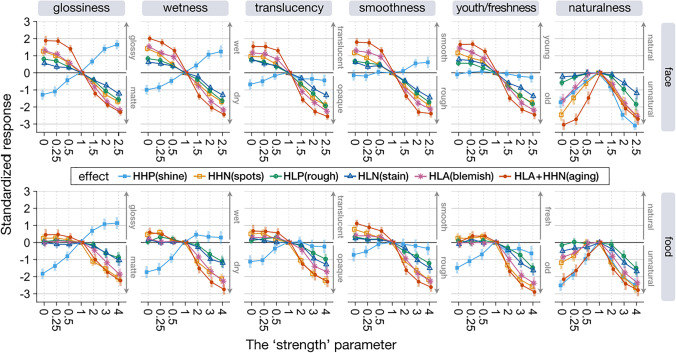
Results of the evaluation experiment. The results of each rating attribute (column) are shown for each image category (face and food). The ratings are standardized with respect to the ratings for unedited images (i.e., images with a strength parameter value of 1). Error bars indicate 95% CI

Figure 11 shows the averaged standardized rating values for each condition (standardization was calculated by subtracting the mean rating value for images with a strength value of 1 from each rating value) and their 95% confidence intervals. The results of the face images are shown in the top row and those of the food images are shown in the bottom row. Each column shows the results for each rating attribute. Each image editing effect is indicated by the color of the line and the shape of the symbol. As an example of how to interpret this graph, editing a face image with a strength value of 0 with the HLA+HHN (aging) effect increases the gloss rating by two points on a six-point scale compared to the unedited image (see the red line in the upper leftmost column in Fig. 11).

To test each material editing effect per rating attribute, the Friedman test (Myles & Douglas, 1973) was conducted for each experimental condition using the *stats::friedman.test* function in R. As responses for the unedited stimulus (strength parameter = 1) were collected twice in each condition, we averaged them before performing the statistical tests. In two conditions, the effect of image editing was not significant (χ^2^(6) = 10.1, *p* = 0.12 for HHP on old–young rating for face, and χ^2^(6) = 9.8, *p* = 0.14 for HHP on rough–smooth rating for food). In all other conditions, each editing effect influenced each rating value (all *p*s < 0.05, corrected for each rating attribute and editing effect with the Bonferroni procedure).

The results show that each editing effect changed the perceived material properties in the expected directions. For example, the HHP effect increased or reduced glossiness and wetness ratings, and reduced the translucency rating. The HHP effect on the smoothness rating was statistically significant only for face stimuli, and its effect on the freshness rating was statistically significant only for food stimuli. The aging (HLA+HHN) effect changed the skin youthfulness rating to old or young depending on the strength parameter value, and changed the food freshness rating to old (but did not increase freshness). The aging effect also affected the other rating dimensions in the same way as observed for the HLA and HHN effects. The HHN (spots), HLP (rough), HLN (stain), and HLA (blemish) are features related to surface roughness and uniformity, all of which affected the smoothness rating. That is, when these features were boosted, the stimulus was rated as rough, and when they were reduced, the stimulus was rated as smooth.

**Fig. 12:**
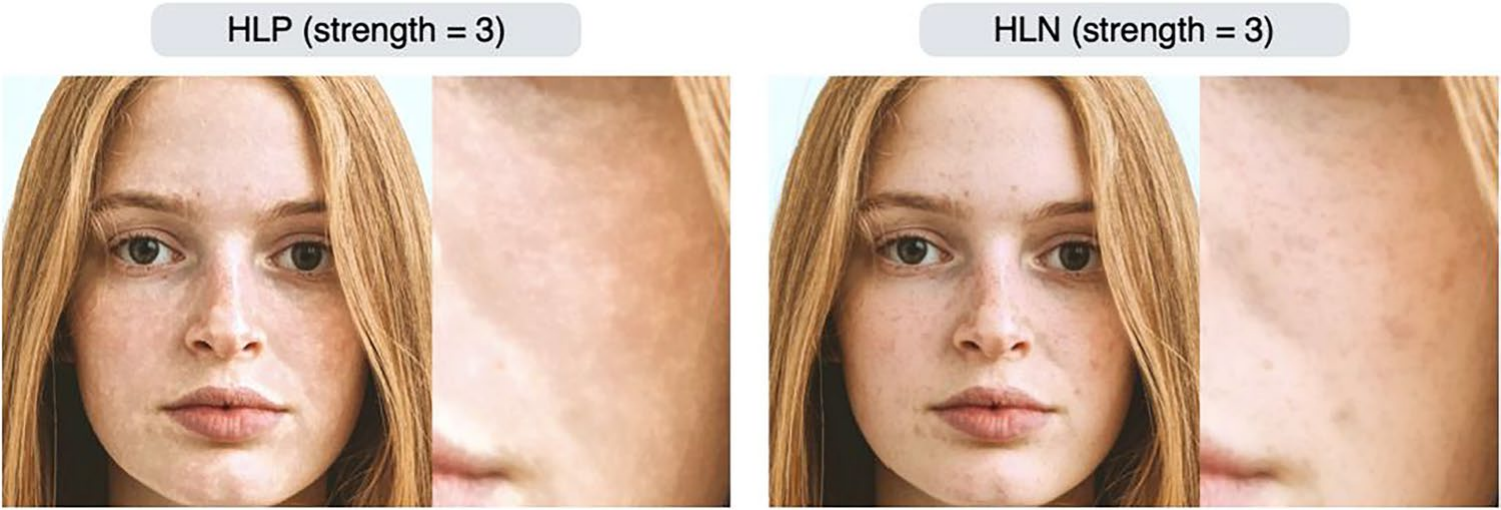
Difference between HLP (rough) and HLN (stain) effects. The image to the right of the output image is a magnified view of the woman’s left cheek

We also found that the roughness-related editing effects (HHN/HLP/HLN/HLA) were also associated with ratings of material properties other than roughness. That is, facial stimuli rated as rough skin were rated as matte/dry/opaque, and facial stimuli rated as smooth skin were rated as glossy/wet/translucent. These results suggest that the HHN/HLP/HLN/HLA effects manipulate image features that are commonly used as perceptual cues across these ratings. Effects such as HLP (rough) and HLN (stain) were rated very similarly (Fig. 11). However, this result does not imply that these editing effects create similar images. In fact, the HLP and HLN effects give different textures to the input image (Fig. 12). Both are similar in that they increase surface roughness, but HLP produces unevenness across the entire surface of the skin, while HLN emphasizes localized dark areas such as stains and blemishes. We believe that the rating scales used in this experiment did not reflect these subtle differences in perceptual experience.

In general, when comparing the results for faces with those for food, the effect of reduce edits (strength parameter value less than 1) on perception was small for food. This may be due to the nature of the image (food) objects used in the experiments: raw meat and oranges, which tend to have a high degree of gloss and translucency. In fact, the glossiness, wetness, translucency, smoothness, and youthfulness/freshness ratings for the unedited (strength = 1) images were all higher for food (4.1 vs. 3.7 for glossiness, 4.3 vs. 3.6 for wetness, 4.0 vs. 3.7 for translucency, 4.2 vs. 3.8 for smoothness, and 4.2 vs. 3.7 for youthfulness/freshness, two sample *t*-test, two sided, all *p*s < 0.05). Thus, perceptual effects of reduce editing on the food images used in the experiment would have been difficult to observe. These results are not considered a procedural artifact (ceiling effect). This is because ratings for stimuli with an intensity parameter of 1 were approximately 4, and there was room for higher ratings.

The results of the naturalness rating showed that the further away from 1 the strength parameter value was, the more unnatural the image was rated (although for some editing effects, such as HLP and HLN, image naturalness was preserved for reducing effects). Therefore, if the naturalness of the image is important, the strength parameter should not be set to an extreme value.

## Limitations

**Fig. 13:**
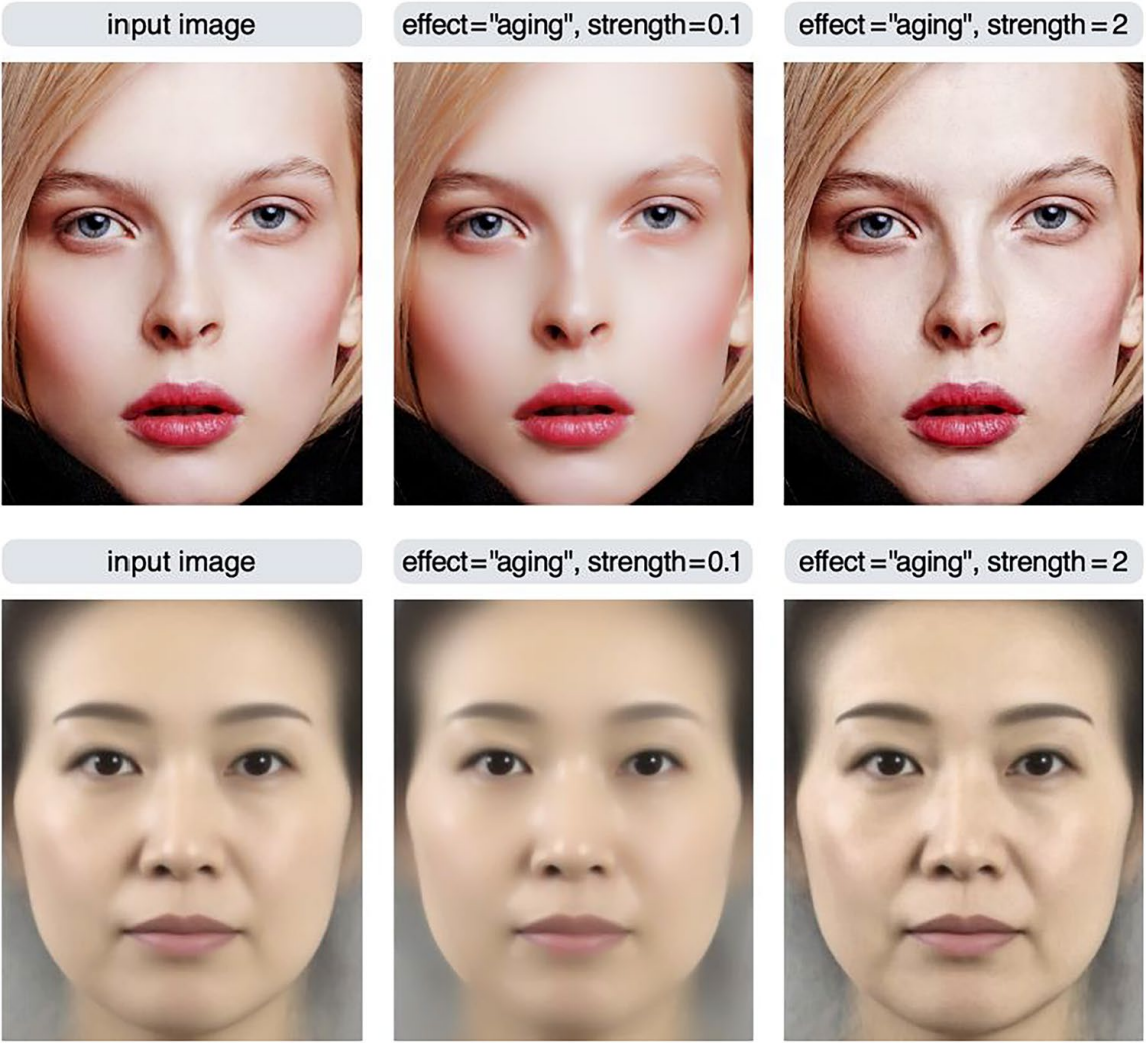
Results of applying the aging effect to a photo of a face (top) and an average face of Japanese females (bottom; adapted from Nakamura et al., 2020). When the skin of the input image is smooth, the aging effect does not work well

This section describes some of the limitations of this package. First, depending on the input image, the material editing effect may not work as intended. If the feature of interest is weak or absent in the input image, the algorithm cannot control the image in that dimension. For example, if a photo of a woman’s face with smooth skin due to heavy makeup or strong lighting is used as an input image, the algorithm cannot increase skin blemishes because it cannot add features that are not present in the input image (Fig. 13). The average face, which is often used in face perception research, also has this limitation because average faces tend to have smooth facial textures.

**Fig. 14:**
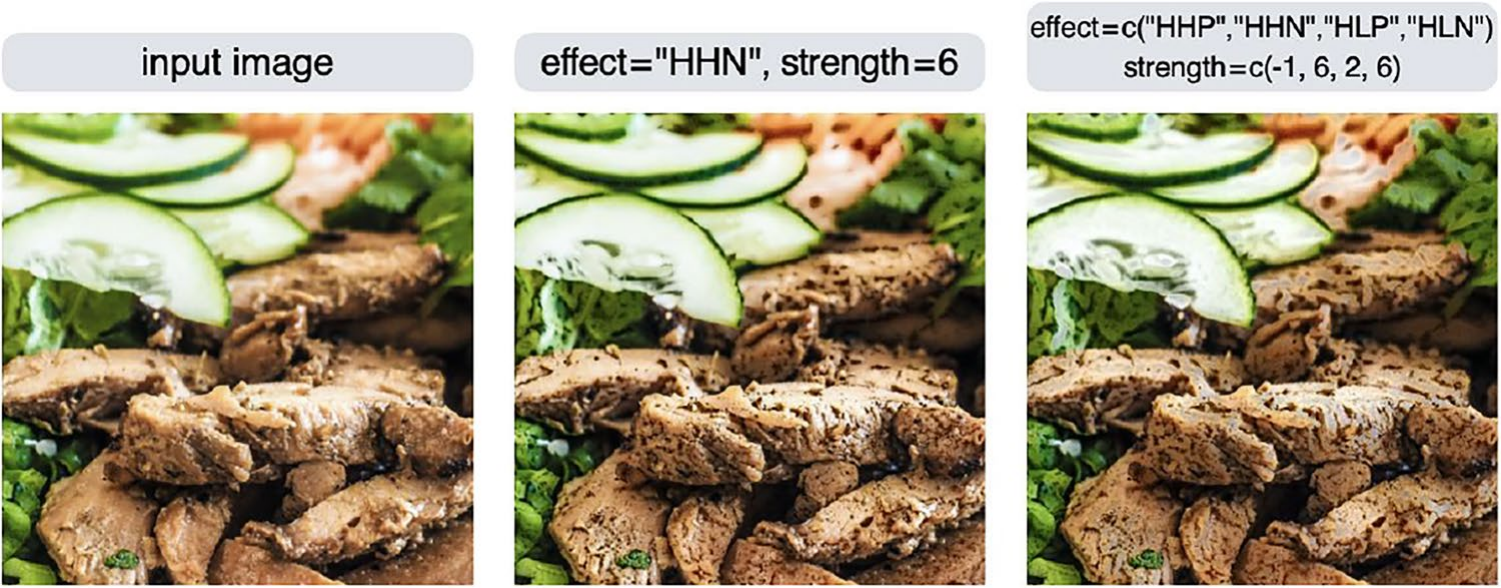
By combining multiple editing effects, it may be possible to create a desired change in appearance. Observe the image at a larger size to see the changes in the image

Figure 14 shows another example where the editing effect is less apparent: the HHN effect is applied to make the food look wilted (middle row), but it does not look wilted enough. One way to deal with this is to increase the strength value, but the larger the value, the more unnatural the image will look. Another way is to apply multiple editing effects (right column). This will increase the spots while concurrently eliminating the gloss, giving the image a more wilted appearance.

Second, the names of effects such as shine and blemish do not necessarily correspond to the appearance of the output image. For example, the effect named “stain” is just an alias for the image feature selection criterion HLN, and the manipulation of the HLN feature is just an adjustment to the lightness information of high spatial frequency, low amplitude, and minus sign in the input image. Therefore, when you want to manipulate stains in an image, it could be more effective to use other effects such as the spots (HHN) effect rather than the stain (HLN) effect. The parameter settings to achieve the desired effect will likely vary depending on the scale of the object and the lighting of the scene, which will be a practical difficulty when using this technique.

Third, the psychophysical properties of the material editing effects are not clear. That is, doubling the strength parameter does not necessarily mean that the perceptual effect will be doubled. It is not a trivial problem to formalize the relationship between the strength parameters and perception, because it will be specific to each material dimension, and possibly to each image category. For example, Boyadzhiev et al. (2015) compared naturalness ratings for face and non-face images and reported that face images are more likely to look unnatural with small image changes. Material perception is subject to complex nonlinearities. As a result, in studies that require a series of controlled images that are perceptually equidistant across each condition, researchers may need to conduct psychophysics experiments to assess the psychophysical properties of the image set.

**Fig. 15:**
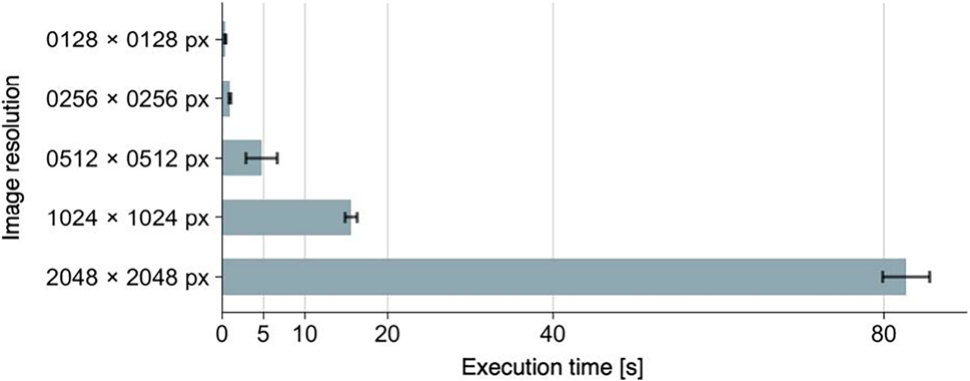
Execution time of the modif function. Ten measurements were taken for each resolution, and the mean and 95% CI of the execution time were calculated. Tested on a MacBook Air (M1, 2020, 8 GB). The type of effect (shine, blemish, etc.) has no effect on the execution time

Finally, it should be noted that the processing time required for image conversion depends on the resolution of the input image. The larger the input image, the more processing time required (Fig. 15). If the resolution of the input image is equal to or smaller than 1024 × 1024 px, the process will be completed in a relatively short time. However, if the resolution of the input image is 2048 × 2048 px, the processing time will be more than a minute (these numbers will also depend on the machine specs). Therefore, the resolution of the input image should be as small as possible, especially when a large number of images need to be prepared. The modif and modif2 functions have an argument named max_size. If the shorter side of the input image is larger than max_size, the image will be automatically scaled down so that the shorter side of the input image matches max_size. The default value of max_size is set to 1280. Thus, if an image with a resolution of 2000 × 3000 px is used as the input image, the output image will have a resolution of 1280 × 1920 px. If you do not want to change the resolution of the image, you can enter a larger value for max_size, or set max_size = NA. This feature was provided to avoid the extremely long execution time when a high-resolution image such as 4K is accidentally used as input.

## Conclusion

In this paper, we presented materialmodifier, an R package for photo editing effects. The software uses image processing techniques to parametrically manipulate the surface properties of objects (e.g., gloss/roughness) in photographs, providing an automatic and reproducible method to create a set of image stimuli. We have confirmed that this software can be used effectively to control the appearance of faces, foods, and objects. We believe that this software will be useful for researchers interested in topics related to material perception, such as face perception and aesthetic evaluation of objects. The package can be installed via CRAN, and documentation and source code are available at https://github.com/tsuda16k/materialmodifier.
